# Extramedullary disease in multiple myeloma

**DOI:** 10.1038/s41408-021-00527-y

**Published:** 2021-09-29

**Authors:** Radhika Bansal, Sagar Rakshit, Shaji Kumar

**Affiliations:** Division of Hematology, Mayo Clinic, Rochester, MN USA 55905

**Keywords:** Myeloma, Chemotherapy

## Abstract

When clonal plasma cells grow at anatomic sites distant from the bone marrow or grows contiguous from osseous lesions that break through the cortical bone, it is referred to as extramedullary multiple myeloma (EMD). EMD remains challenging from a therapeutic and biological perspective. The pathogenetic mechanisms are not completely understood and it is generally associated with high-risk cytogenetics which portends poor outcomes. There is a rising incidence of EMD in the era of novel agents, likely a reflection of longer OS, with no standard treatment approach. Patients benefit from aggressive chemotherapy-based approaches, but the OS and prognosis remains poor. RT has been used for palliative care. There is a need for large prospective trials for development of treatment approaches for treatment of EMD.

## Introduction

Multiple Myeloma (MM) is defined by the presence of ≥10% clonal bone marrow (BM) plasma cells (PC) associated with features of hypercalcemia, renal failure, anemia or lytic bone lesions or the presence of biomarkers such as ≥60% BMPC, involved to uninvolved FLC ratio ≥100, or the presence of ≥2 marrow lesions on MRI [1]. Despite high-dose chemotherapy with stem cell support (HDT) and novel therapeutic agents, prognosis remains poor. When a sub-clone of PCs is able to grow outside of marrow, it results in development of disease outside the marrow, termed as extramedullary multiple myeloma (EMD).

## Classification

The term extramedullary can be confusing and as there is a lack of consensus regarding the classification, we put forward a convenient way to classify them in a manner that reflects the prognosis and the therapeutic approach (Table 1) [1–6]. EMD can be present either at initial diagnosis (primary EMD) or at relapse (secondary EMD) [3, 7].

**Table 1:** Classification of EMD.

	Type of EMD	Definition
a.	Solitary Plasmacytoma (SP) with no marrow involvement	Biopsy-proven bone or soft tissue lesion with evidence of clonal plasma cells. However, marrow has no clonal PCs and no additional abnormality on imaging and absence of CRAB criteria.
b.	Solitary plasmacytoma with minimal marrow involvement	SP with <10% clonal BMPC
c.	Bone associated EMD with MM (EMM)	Soft tissue mass arising from bone lesions and growing contiguously
d.	Bone independent EMD with MM (EMM)	Isolated extra-osseous plasma cell tumors not contiguous with bone lesions
e.	Organ infiltrating EMD	CNS myeloma, diffuse liver involvement etc
f.	Plasma cell leukemia (PCL)	Traditionally, this aggressive variant of MM was defined by the presence of circulating PCs (>20% and/or absolute count >2 × 10^9^/L). However, this criteria was updated recently by including those with ≥5% cPCs or an absolute number ≥0.5 × 10^9^ cells/L detected morphologically on a peripheral blood smear [5]. The corresponding quantitative cutoff for circulating PCs was determined as 200 cPCs/µl on multiparametric flow cytometry [8].

The symptoms due to EMD are typically related to the site of lesions—a summary of literature regarding sites involved in EMD is provided in Table 2.

**Table 2:** Summary of sites involved in EMD with presentation and treatment options.

Site involved	Presentation	Incidence	OS	Treatment options	Ref
CNS- Brain parenchyma or meninges	Lethargy, nausea or vomiting, headache, confusion, paresthesia or seizures; visual, gait, and speech disturbances	3%	1 month	Whole brain radiation therapy, intrathecal chemotherapy, and systemic chemotherapy	[46]
Skull	Smooth, firm, and non-tender mass on skull	<1%		High-dose dexamethasone	[3]
Orbit	Generally unilateral soft tissue orbital mass with complaints of headache, proptosis which is painless in nature, decreased vision, diplopia, restriction of eye movement and swelling, corneal crystalline deposits	<1%	28 months	Local excision as a salvage surgery, whole brain radiation therapy, intrathecal chemotherapy, and systemic chemotherapy	[47]
Vertebrae	Spinal cord or root compression, back pain	<1%	–	RT, intrathecal chemotherapy	[48]
Breast	Breast lump ranging from 1 to 7.5 cms	9% in primary EMD and 3% in secondary EMD	–	Surgical excision with adjuvant RT. Chemotherapy should be considered for tumors greater than 5 cm, high grade tumors and patients with refractory and / or relapsed disease. SCT.	[11, 35, 49]
Thyroid	Painful swelling on the side of the neck accompanied with odynophagia, dysphagia, and hoarseness	2.9%	–	Chemotherapy with or without autologous SCT. External beam RT –when organ function loss is contemplated post-surgery.	[50–52]
Soft tissue of neck	Soft tissue swelling in the neck, unilateral nasal obstruction, more common in males, associated with epistaxis, facial swelling, pain and rhinorrhea. Can also present with headache, ptosis, diplopia, CN palsies II, III, IV, VI is sphenoid sinus is involved	10%	–	Tumor size <5 cm-RT 30-40 Gy #20 Tumor size >5 cms- RT 40-50 Gy. Chemotherapy is considered if tumor size >5 cm, high-grade tumor, refractory/relapsed disease. Surgical excision may be considered.	[25, 26, 53, 54]
Lungs	Unilateral Pleural effusion (right>left), pulmonary nodule, hilar mass, with atypical symptoms. Can have concurrent ascites	2.65%	2.8–4 months	Intrapleural bortezomib biweekly during induction and weekly or fortnightly during consolidation and maintenance along with systemic chemotherapy, concurrent pleurodesis or ICD drainage	[55]
Spleen	Silent course, incidental finding on autopsies, can rarely present with left upper quadrant pain, painful splenomegaly, rarely splenic rupture	9% in primary EMD and 11.5% IN secondary EMD	–	Splenectomy	[11,56]
Heart	Male preponderance, presents with dyspnea, tachycardia, pericardial effusion with or without tamponade, distant heart sounds, distended neck veins and positive kussmaul sign, pericardial or atrial mass	0.4%	13.5 weeks	Pericardial window for drainage, chemotherapy +/− high dose cortocosteroids	[57]
Liver	Hepatomegaly, jaundice, ascites, and fulminant liver failure, mildly elevated liver transaminase levels	28.8%	–	Systemic chemotherapy	[56]
Pancreas	Decreased appetite, worsening peri-umbilical discomfort, pulsatile abdominal mass, bilateral rib pain, jaundice, homogeneous solid mass on CT	2.3%	7 months	Systemic chemotherapy such as with VRD, RT, SCTation	[58]
Gatro-intestinal tract	Non-specific gastrointestinal symptoms, including anorexia and weight loss, abdominal pain, vomiting, and, rarely, gastrointestinal bleeding, usually from an ulcerated lesion. small bowel is the most common site of involvement, followed by stomach, colon, and esophagus.	<5%	–	RT or surgery along with systemic chemotherapy	[59]
Omentum	Ascites, generally an autopsy finding	–	1.5 months	–	[60]
Testis	Testicular swelling, erythema, pain may or may not be present	0.1%		Radical orchiectomy	[61]
Skin	Centrifugal appearance of multiple erythematous nodules or papules, or plaques that show a nodular or diffuse interstitial pattern.	1.14%	0.4–108 months (8.5 months)	Chemotherapy, RT, SCT	[62]
Subcutaneous tissue	Single or multiple large highly vascularized subcutaneous nodules with a red-purple appearance	0.6%	–	bortezomib-containing regimen followed by ASCT	
Lymph node	Non-tender, enlarged lymph nodes. Weight loss maybe present. Most common site- paratracheal lymph node	23.1%	–	–	[56]
Muscle	Symmetric proximal muscle weakness and tenderness	4.5%	–	Systemic chemotherapy	[11]
Female reproductive system	Pelvic pain, profuse menorrhagia, and severe anemia	–	–	total abdominal hysterectomy with bilateral salpingo-oophorectomy	
Adrenal glands	Incidental finding on imaging or autopsies	7.7%	–	Surgical excision	[56]

- insufficient data.

## Epidemiology

Overall incidence of EMM is 13%:7% at diagnosis and 6–20% at relapse [8]. 85% of these are bone-associated and the median age for patients is higher as compared to patients with bone-independent EMD (71 vs 60.5 years) [2]. There has been an overall increase in the incidence of EMM from 6.5% in 2005 to 23.7% in 2014 [9]. Median time from diagnosis to occurrence of EMM has been observed to be 19–23 months [2, 8]. The results of total therapy protocol trials also reported that extra medullary involvement at presentation was more common among those with high-risk translocations t(14;16) and t(14;20) and was associated with poor overall survival (OS) [10].

Patients with osteolytic lesions and hypercalcemia are at a higher risk for developing EMD. Other significant risk factors include therapeutic history (>2 lines of treatment ± treatment duration >6 months) and allogenic SCT (auto-allo-SCT) [11, 12]. It is quite possible that the increasing frequency of EMD at relapse among patients with MM reflects the improved OS in general and that we are seeing a phase of the disease we did not reach before the advent of newer therapies.

## Pathogenesis

**Fig. 1:**
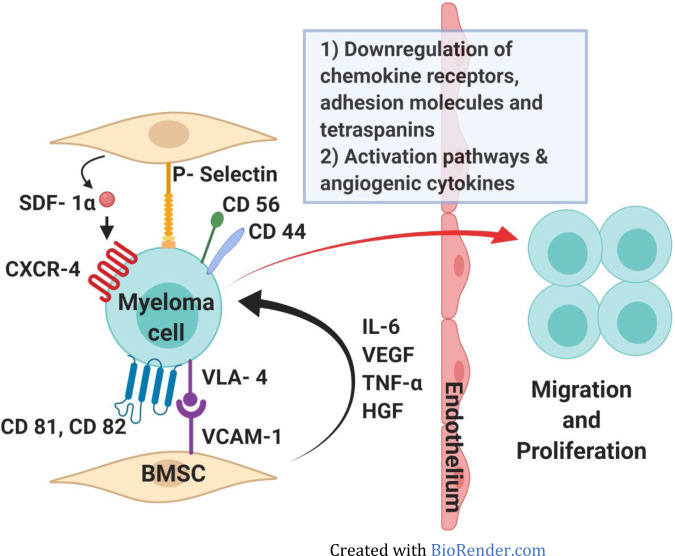
Pathogenesis of extramedullary spread in multiple myeloma. SDF-1- Stromal cell derived factor-1, CXCR-4- Chemokine receptor type 4, VLA-4- Very late antigen-4, VCAM-1- Vascular cell adhesion protein-1, VEGF- Vascular endothelial growth factor, TNF-a- Tumor necrosis factor- alpha, HGF- Hepatocyte growth factor, IL-6- Interleukin-6.

The interaction between myeloma cells and the BM microenvironment activates signaling cascades and mediates chemotaxis and adhesion of myeloma cells to BM (Fig. 1). The adhesion is augmented by binding of stromal-derived factor 1 a (SDF 1-A) to CXCR4 receptor and adhesion molecules like VLA-4, P-selectin, CD 56, and CD 44 [13]. Tumor dissemination occurs due to (i) low expression of chemokine receptors and adhesion molecules [4], (ii) underexpression of membrane-embedded CS81/CD 82 tetraspanins [14] and overexpression of tumor promoter heparanase enzyme, (iii) upregulation of CXCR4 by various growth factors and hypoxic conditions in tumor microenvironment [15] and acquisition of EM phenotype regulated by CXCR4 [15, 16]. A possible PCAT-1/Wnt β-catenin signaling axis has also been implicated in growth, OS, and migration of MM cells [17, 18]. Head and neck and liver have been reported as the most common location at diagnosis followed by pleural fluid at relapse [19]. It was hypothesized that specific tropism or homing of EMM clones makes them more prone to trafficking to these sites.

Recent studies have revealed that long non-coding RNA like MALAT1 and MEG-3 regulate gene expression at the transcriptional, post‐transcriptional, and epigenetic levels and are involved in tumor initiation, metastasis, and drug resistance [20]. MALAT1 located on chromosome 11 was observed to be markedly higher in EMD as compared to intramedullary MM cells [21]. It was observed that patients with a greater decrease in MALAT1 after initial treatment had a significantly prolonged progression‐free survival (PFS) duration, while patients with smaller MALAT1 changes after treatment had a significantly higher risk of early progression [21].

## Immunophenotype

Studies have shown that EMDs have a higher proliferative index, lower p27 expression, and CCND-1 and p53 co-positivity [22]. BCl-2 and Bcl-xl are strongly positive, CD56 is downregulated and CD44 is upregulated [22, 23]. Immuno-phenotyping helps not only in identifying the cell but also in establishing the correct diagnosis.

## Cytogenetic profile

Genetic aberrations in myeloma are usually identified using Fluorescence in-situ hybridization (FISH) and have an important prognostic value in MM. However, cytogenetic features of EMD are not well defined in literature. A few studies have reported association of high-risk cytogenetics like t(4;14), t(14;16), gain(1q21), and del(17p) in patients with EMD [2, 24, 25]. Studies have also identified del(17p13) and del(13q14) as markers for progression to EMD [2, 26] and del(13) as risk factor for EM relapse. Gain(1q) was associated with inferior outcome [27]. High risk cytogenetics was more frequent in patients with organ involvement (47%) vs EMM [28].

## Clinical evaluation

Along with the routine myeloma workup, EMD requires a tumor biopsy/FNAC for immune-histochemistry (Table 3) and a BM biopsy to evaluate PC morphology and the degree of total PC infiltration [29]. Patients who develop EM spread during their disease course have significantly lower levels of serum M-protein and hemoglobin and significantly higher levels of lactate dehydrogenase (LDH) than those who present with EMD at diagnosis [8]. Using sensitive imaging techniques including MRI and PET/CT, EMD may be found in up to 30% of MM patients across the overall disease course.

**Table 3:** Recommended workup for EM multiple myeloma.

Diagnostic tools	Comments
Laboratory	Complete blood count with differential, peripheral smear
	Chemistry—Creatinine, albumin, corrected calcium
	Lactate dehydrogenase
	Beta-2 microglobulin
	Serum quantitative immunoglobulins
	Serum protein electrophoresis
	Serum Free Light Chain (FLC) assay
	Urine—24-h urine for total protein, urine protein electrophoresis
Bone marrow aspiration and biopsy	If no plasma cells are detected—SP with no marrow involvement
	If <10% plasma cells are detected—SP with minimal marrow involvement
	FISH if plasma cells identified
Tumor biopsy/sampling	Usually sheets of plasma cells, identifiable by morphology
	IHC if required for light chain restriction
	Ki67 stains can help determine proliferation rate
	FISH, mutation panel (if applicable)
Radiology	Skeletal survey
	18 Fluorodeoxyglucose positron emission tomography (FDG-PET)
	Computed tomography (CT), Magnetic resonance imaging (MRI)
Multiparameter flow cytometry	True solitary plasmacytoma—characterized by flow-negative bone marrow and absence of M protein
	Circulating plasma cells >200 cPCs/µL - PCL

## Treatment

### EMM

#### Radiotherapy (RT)

There is no consensus on use of RT in EMM except for SP. A few cases have reported the use of RT with good outcomes in EMD as outlined in Table 2.

#### Induction chemotherapy

With a rising incidence of EMD in the era of novel agents, it was hypothesized that newer drugs lead to drug resistant, inherently aggressive, and BM-independent clones [7]. However, there is no clinical evidence supporting the same [30]. Superior complete response rates in de-novo EMD patients have been reported with novel agents (thalidomide, lenalidomide and bortezomib-based regimens) vs conventional chemotherapy [31] (Table 4). In relapsed/refractory (r/R) patients with EMD, lymphoma-like polychemotherapy regimen such as PACE (cisplatin, doxorubicin, cyclophosphamide, and Etoposide), Dexa-BEAM, and HyperCVAD (hyperfractionated cyclophosphamide, vincristine, doxorubicin, and dexamethasone) followed by ASCT (ASCT) or auto-allo-SCT have been successful [32, 33]. Newer generation IMiDs such as pomalidomide have also been effective at the time of relapse [7]. Carfilzomib is also active but has inferior outcomes in bone-independent EMD compared to bone-related EMD [34]. One should consider the previous lines of therapy and the duration of response at relapse.

**Table 4:** Summary of studies evaluating treatment options for EMD.

Author	Patient group	Treatment arm (% of patients)	Type of EMD	Complete response rate (%)	Median PFS (months)	Median OS (months)	Limitation of study	Ref
Gagelmann et al. [28]	Newly diagnosed MM with EMD (488)—40% with high risk cytogenetics	Bortezomib-based induction (73)		21	4 year PFS-42%	4 year OS-69%	Absence of data on maintenance therapy, salvage treatment, or details on induction therapy beyond whether bortezomib was used or not	[37]
		Non-bortezomib-based induction (27)		17	4 year PFS-34%	4 year OS-64%		
		First line ASCT (77)			4 year PFS-43%	4 year OS-70%		
		Tandem ASCT (17)			4 year PFS-52%	4 year OS-83%		
		Auto–allogeniec transplant (6)			4 year PFS-58%	4 year OS-88%		
Beksac et al. [46]	Newly diagnosed EMD (130/226)	Initial therapy—IMiD-based (74.7%)/ PI-based (10%) followed by ASCT (51.5%)	Bone-independent MM	19.3	38.9	46.5	Selection bias—age < 45 not included	[56]
			Bone-associated MM	34.2	51.7	N.R.		
	EMD at relapse (96/226)	Initial therapy—IMiD-based (10.4)/ PI- based(41.7%) followed by ASCT (4.1%)	Bone-independent MM	9	13.6	11.4		
			Bone-associated MM	54.5	20.9	39.8		
Gagelmann et al. [9]	Adult patients with EMD at diagnosis who received single ASCT within 12 months of diagnosis or a tandem ASCT within six months from first ASCT as first line therapy (682/3744)	Pre-ASCT	Bone-independent MM	11.7	N.R. (3 year PFS-59.8%)	N.R. (3 year OS-83.6%)	Selection bias—elderly patients not transplanted are not included	[10]
			Bone-associated MM	21.5				
		Post-ASCT	Bone-independent MM	36.1	24	N.R. (3 year OS-58%)		
			Bone-associated MM	41.6	36	N.R. (3 year OS-77.7%)		
		Post-tandem ASCT	Bone-independent MM		N.R. (3 year PFS-56.2%)	N.R. (3 year OS-52%)		
			Bone-associated MM		N.R. (3 year PFS-59.4%)	N.R. (3 year OS-82.6%)		
Kumar L et al. [31]	EMD at diagnosis or prior to ASCT (44/271) with 200 mg/m^2^ melphan conditioning	Initial therapy- Novel agents (52.3%)	EMD	52.2% (12/23)	18	32	Small sample size. Lack of cytogenetic data.	[42]
		VDD and alkylating agents (47.7%)		9% (2/21)				
Shin et al. [24]	EMD at diagnosis or prior to ASCT with 88.2% patients receiving 200 mg/m^2^ melphan conditioning (93/239)	Initial Therapy- TCD (34.5%)/ VAD (27.6%)/ RT (51.7%)	Bone-independent MM	31	12	37		[33]
		Initial Therapy- TCD (29.7%)/ VAD (37.5%)/ RT (45.3%)	Bone- associated MM	40.6	28	67		
Gozzetti et al. [35]	Intra cranial-MM (50)	Autologous/ allogenic SCT- (24%)	CNS EMD and osteodural EMD	50%	34	46		[63]
		Chemotherapy- novel and old agents (72%)			5	12		
		RT (32%)			12	25		
Short et al. [7]	EMD in relapsed refractory MM (13/174) Patients had prior exposure to bortezomib (78%), IMiD agents -thalidomide or lenalidomide (100%) before the diagnosis of EMD	Pomalidomide plus low-dose dexamethasone in phase II clinical trial	Primary bone-independent MM	15.4		16	Only included bone-independent MM. Bone-associated MM were excluded	[7]
			Treatment-emergent Bone-independent MM					

*PFS* Progression free survival, *OS* Overall Survival, *N.R.* Not Reached, *TCD* [thalidomide, cyclophosphamide, and dexamethasone, *VAD* [vincristine, adriamycin, and dexamethasone, *VDD* [vincristine, doxorubicin, and dexamethasone, *ASCT* Autologous stem-cell transplant.

Extramedullary tumor masses in CNS most frequently arise from bone lesions in the cranial vault, skull base, nose, or paranasal sinuses, whereas primary dural (pachy-meningeal) involvement is rare. The OS with osteodural involvement (25 months) is three times more than leptomeningeal involvement (6 months) [35]. For CNS EMD, a combination of CNS directed treatment including RT and IT chemotherapy and systemic therapy including novel agents which can cross blood brain barrier (BBB) has shown activity [35, 36]. IMIDs are more likely to cross BBB than PIs and more prospective data is needed to determine ideal strategy [37].

There is paucity of data with use of Daratumumab in EMD. An updated pooled analysis of studies (GEN501 part 2 and SIRIUS) evaluating role of daratumumab in heavily pre-treated patients reported an overall response rate (ORR) in subset of patients with EM involvement was 16.7% (95% CI: 3.6–41.4) with improved OS in responders/minimal response/stable disease [38]. There are also several case reports with response to daratumumab in EMD.

Innovative approaches using adoptive cell therapies (chimeric antigen receptor T cells) have recently shown promising results in a limited number of relapsed patients with EMD [39]. In a meta-analysis on BCMA CAR-T cell therapy, the presence of EM disease at time of infusion was not associated with lower response rates showing a pooled response rate of 78% vs 82% overall [40]. The high response rates with anti-BCMA CAR-T therapy despite EM disease demonstrate the need for more focused subgroup analysis in upcoming CAR-T studies.

#### SCT

The preferred next step in patients who respond to induction therapy is transplant. However, the benefit of ASCT in patients with EMD appears to be more limited. The Spanish PETHEMA group observed a significantly shorter median OS (46.7 months vs NR) but no significant difference in 2-year PFS after ASCT with high-dose melphalan conditioning. The poor outcome after single ASCT can be attributed to high-risk cytogenetics which can be found in almost 40% patients with EMM. Single vs multiple sites of EMD as well as organ involved can also impact prognosis after ASCT [9]. Upfront tandem transplant has been shown to overcome poor outcomes in these patients compared to single ASCT [28]. Studies evaluating tandem transplantation suggest high-risk subgroups, including patients failing to achieve VGPR after single ASCT, International Staging System (ISS) stage II/III, and high-risk cytogenetics, may benefit most from tandem transplantation [41]. However, a EBMT registry study reported similar 3-year PFS and OS with both first-line tandem and single ASCT in patients with EMD [9, 42]. (Table 4).

#### Relapse after transplant

Patients with MM with EMD at diagnosis or during the disease course have a higher risk of EMD at relapse following HDT. The relapse rate is generally similar between bone-independent MM and bone-associated MM [24]. Various sites like bone, abdomen, and chest have been reported to be involved at the time of relapse [19, 24]. Although the mechanism is largely unclear, but worsening disease status at time of transplant may enhance the risk of EMM [43].

Gagelman et al. reported cumulative incidence of relapse in NDMM patients with EMD as 54% after single ASCT, 47% after tandem ASCT, and 30% after auto–allogeneic transplant [28]. Even though allo-SCT is associated with long-term disease-free OS, it is associated with high transplant-related mortality. A higher incidence of EM relapse (45–55%) has been observed with auto-allo-SCT with reduced intensity conditioning (RIC) [44]. A German study used auto-allo-SCT either as first line treatment or at the time of relapse as the escalation approach. They reported relapse in 49% of the patients with EMD present in one-third of the cohort. OS in EMD group was significantly inferior as compared to intramedullary relapse [45]. Allo-SCT takes advantage of a tumor cell-free graft along with the graft-versus-myeloma (GVM) effect targeting residual malignant plasma cells. Furthermore, allo-SCT allows for donor lymphocyte infusions as an additional intervention that has shown remarkable responses, clearly demonstrating the intensification of a GVM effect. Hence, in patients requiring rescue therapy, allo-RIC should be considered as a platform for additional therapeutic strategies after transplantation to take advantage of the GVM effect.

#### Prognosis

EM involvement is one of the indicators of poor prognosis in MM, with high mortality and an average OS time of 36 months [10, 35]. Factors causing worse progression-free OS and OS: (a) EMD, (b) EMD at relapse, (c) bone-independent EMD with MM, (d) multiple organ involvement, (e) CNS involvement, (f) No ASCT, (g) not achieved complete response post-SCT, (h) β2-microglobulin >5 mmol/L, (i) ISS II & III (j) acute GVHD [9, 31, 35, 44, 46]. On multivariate analysis, Shin et al. also reported platelet counts as predictive of poor PFS and bone marrow plasma cell percentage as predictive for poor OS after ASCT [24].

#### Cause of death

The EBMT report on EM multiple myeloma observed non-relapse mortality (NRM) at three years in 3% patients with bone associated EMD, and 7% in patients with EM organ involvement. The main causes of death were relapse or progression (86.3%), infection (7.1%), secondary malignancy or post-SCT lymphoproliferative disorder (3.6%), organ damage or failure (1.8%) and toxicity (0.4%) [9].

## Future considerations

EMD presents a spectrum of disease presentations in MM with ill-defined boundaries. There is an urgent need for consensus on criteria defining EMD. The incidence of EMD is largely underestimated due to lack of prospective studies on large cohorts. New guidelines should be formulated which provide algorithms for treatment and follow-up of EMD using RT, chemotherapy, and surgery considering category, location, and tumor size. Large, randomized multi-center studies with long follow up are required to assess the efficacy and safety of available treatment options. Newer drugs like monoclonal antibodies, immunotherapy, and BCL-2 inhibitors are also worth exploring.
